# Supplementary material for: Single-cell transcriptomic analysis of the tumor ecosystems underlying initiation and progression of papillary thyroid carcinoma
Source: Nat Commun. 2021 Oct 18;12:6058. doi: 10.1038/s41467-021-26343-3 (PMC8523550; doi:10.1038/s41467-021-26343-3)
Supplement: Supplementary file 1 — Supplementary Information [file 41467_2021_26343_MOESM1_ESM.pdf]

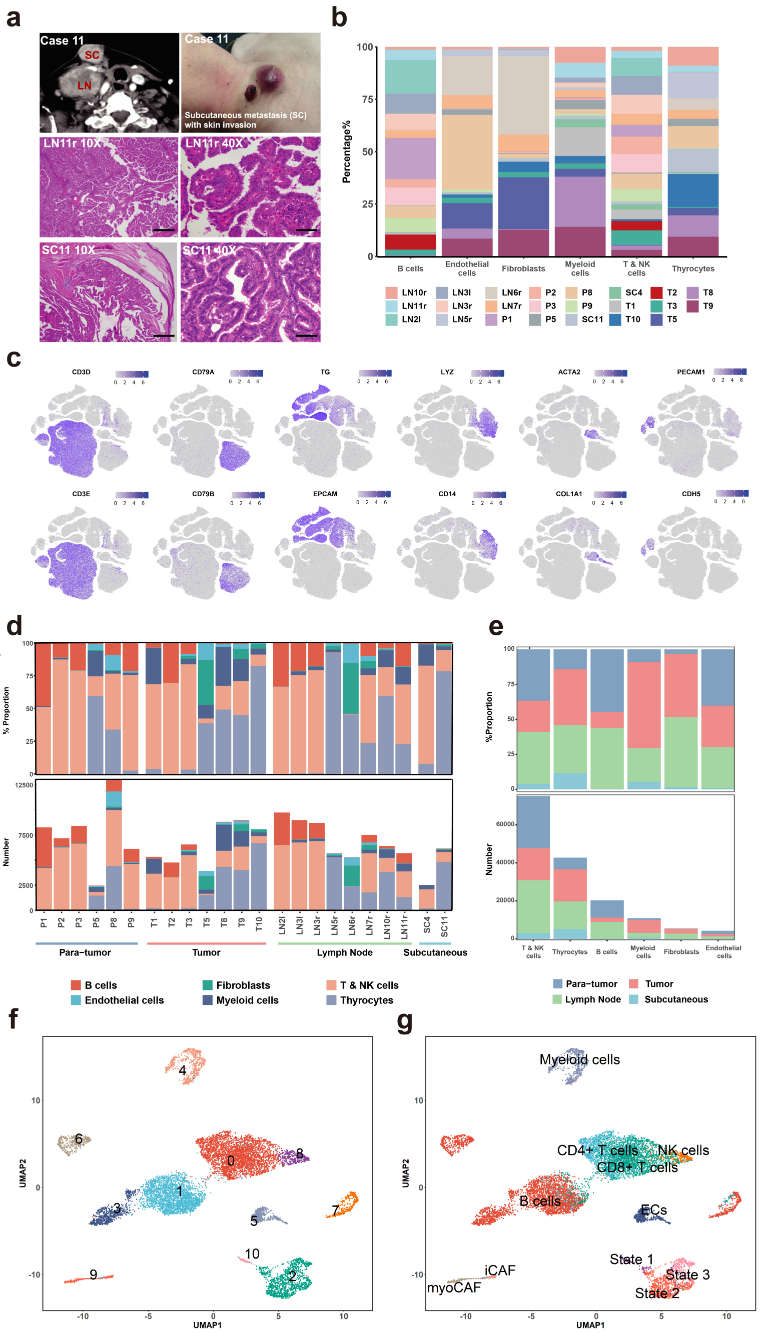

Supplementary Figure 1. Basic Information of the scRNA-seq Data. a, Clinical presentation, H&E staining and CT scans of two samples (SC11 and LN11r) from Case 11. Three independent experiments were performed and generated similar results. Scale bar (10X) = 200 μm; scale bar (40X) = 50 μm. **b**, Contribution of the 23 samples to the six major cell lineages. **c**, Expression level of canonical marker genes for each major cell lineage. **d**, Number and proportion of each major cell lineage in the 23 samples. **e**, Number and proportion of cells from different tissue types for each major cell lineage. **f-g**, Validation of the major cell types in our study using a public PTC scRNA-sequencing dataset (GSE158291). Source data are provided in the Source Data file.

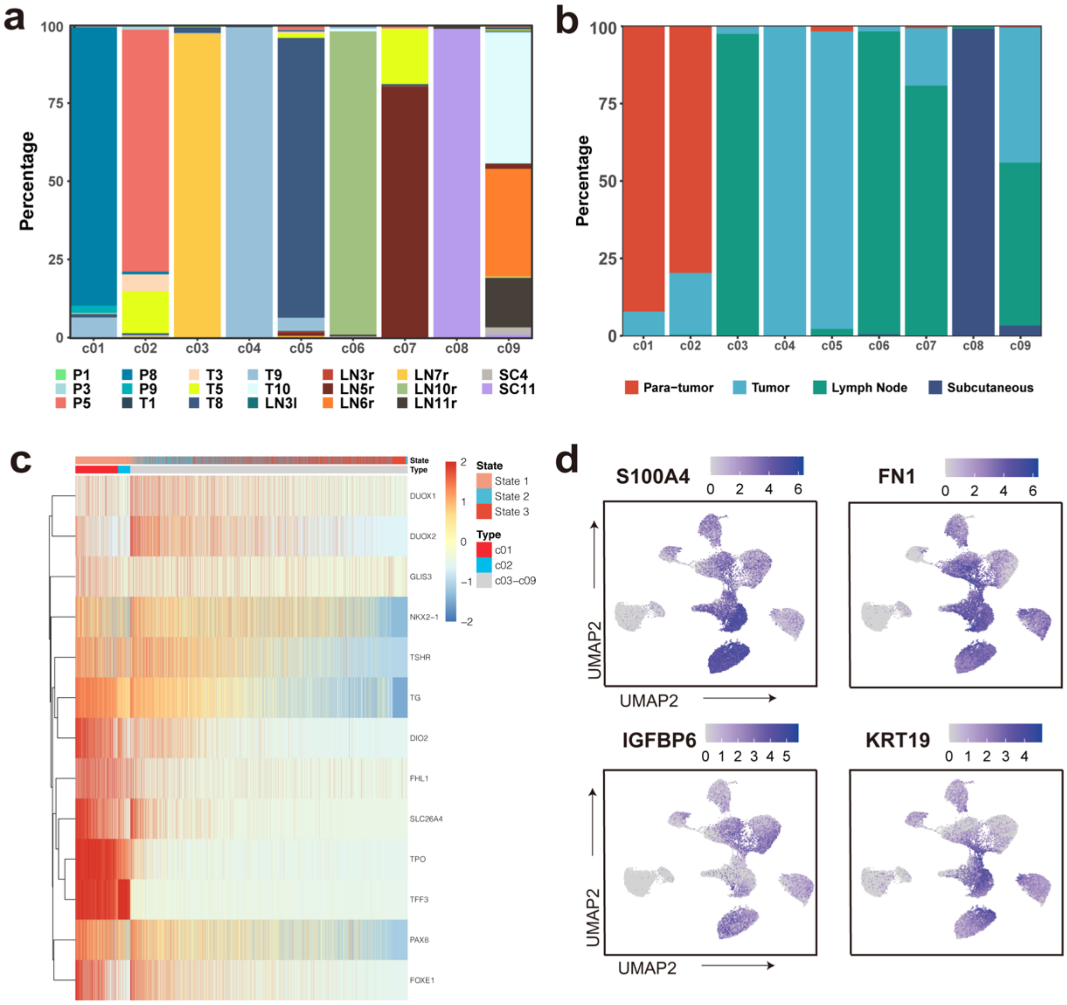

Supplementary Figure 2. Clustering of the Thyrocyte Lineage. a, Fractions of cells from different samples for each thyrocyte cluster. **b**, Fractions of cells from different tissue types for each thyrocyte cluster. **c,** Heatmap showing the expression level of TDS genes in different cell types and states. **d**, Expression levels of canonical thyroid differentiation-related genes and PTC-related genes in these thyrocyte clusters. Source data are provided in the Source Data file.

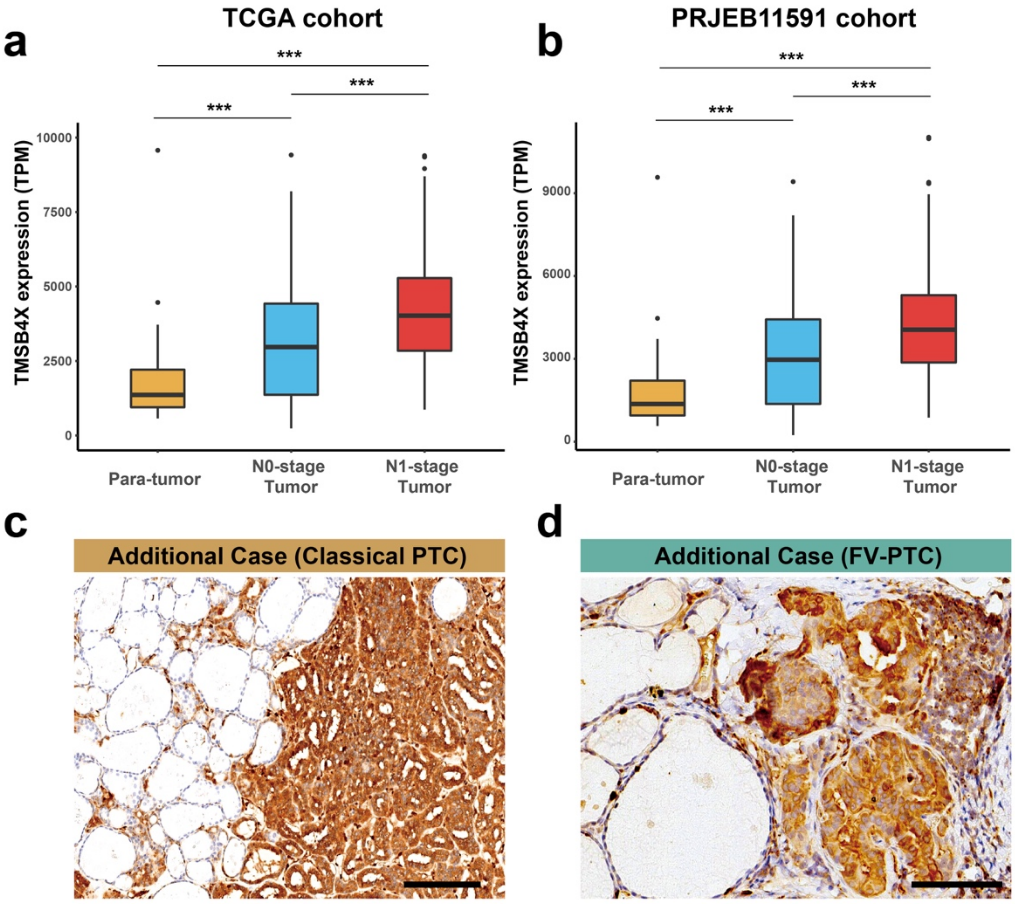

Supplementary Figure 3. Expression Profiling of *TMSB4X* in PTCs. a, Expression level of *TMSB4X* increases continuously from para-tumors (n = 56) to N0-stage tumors (n = 223) and to N1-stage tumors (n = 219) of PTCs in TCGA cohort. **b,** Expression level of *TMSB4X* increases continuously from para-tumors (n = 40) to N0-stage tumors (n = 68) and to N1-stage tumors (n = 35) of PTCs in PRJEB1151 cohort. **c-d**, IHC staining of TMSB4X protein in two additional cases of (**c**) classical PTC and (**d**) FV-PTC. Scale bar = 200 µm for (**c**) and scale bar = 100 µm for (**d**). Three independent experiments were performed and generated similar results. In (**a**) and (**b**), The middle lines of the boxplots show the median (central line), the lower and upper hinges show the 25-75% interquartile range (IQR), and the whiskers extend from the hinge to the farthest data point within a maximum of 1.5x IQR. Two-sided Wilcoxon rank sum tests were performed in (**a**) and (**b**) without multiple adjustment; ***$P < 0.001$. Source data are provided in the Source Data file.

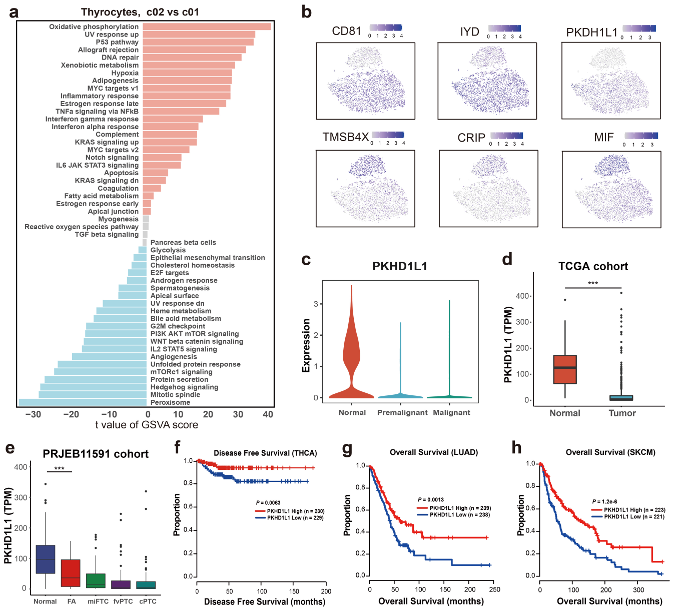

Supplementary Figure 4. Key Pathways and Marker Genes between Normal and Premalignant Thyrocytes. a, Differences in pathway activities between the two non-malignant thyrocyte subtypes (c02 versus c01) by GSVA. **b,** t-SNE projection showing the top DEGs between the c01 and c02 clusters. **c**, Violin plot showing decreased expression of *PKHD1L1* in premalignant and malignant thyrocytes compared with that in normal thyrocytes. **d**, Boxplot showing decreased expression of *PKHD1L1* in tumor tissues (n = 491) compared with that in normal thyroid tissues (n = 56) in the TCGA cohort. Two-sided Wilcoxon rank sum tests were performed without multiple adjustment; ***P < 0.001. **e**, Boxplot showing a downward *PKHD1L1* expression with the decline of follicular patterns across different histologic subtypes (normal (n = 81), FA (n = 25), miFTC (n = 30), fvPTC (n = 48), cPTC (n = 77)) in the PREJEB11591 cohort. Two-sided Wilcoxon rank sum tests were performed without multiple adjustment; ***P < 0.001. **f**, *PKHD1L1*-high (n = 230) and *PKHD1L1*-low (n = 229) PTCs showed a significant difference in disease-free survival time in the TCGA cohort. **g-h**, Kaplan-Meier plots for overall survival of *PKHD1L1*-high (n = 239) and -low (n = 238) in lung adenocarcinoma (LUAD) patients **(g)**, and overall survival of *PKHD1L1*-high (n = 223) and -low (n = 221) in skin cutaneous melanoma (SKCM) patients **(h)** from the TCGA database. In (**f-h**), Log-rank test (two-sided) was performed. In (**d**) and (**e**), The middle lines of the boxplots show the median (central line), the lower and upper hinges show the 25-75% interquartile range (IQR), and the whiskers extend from the hinge to the farthest data point within a maximum of 1.5x IQR. Source data are provided in the Source Data file.

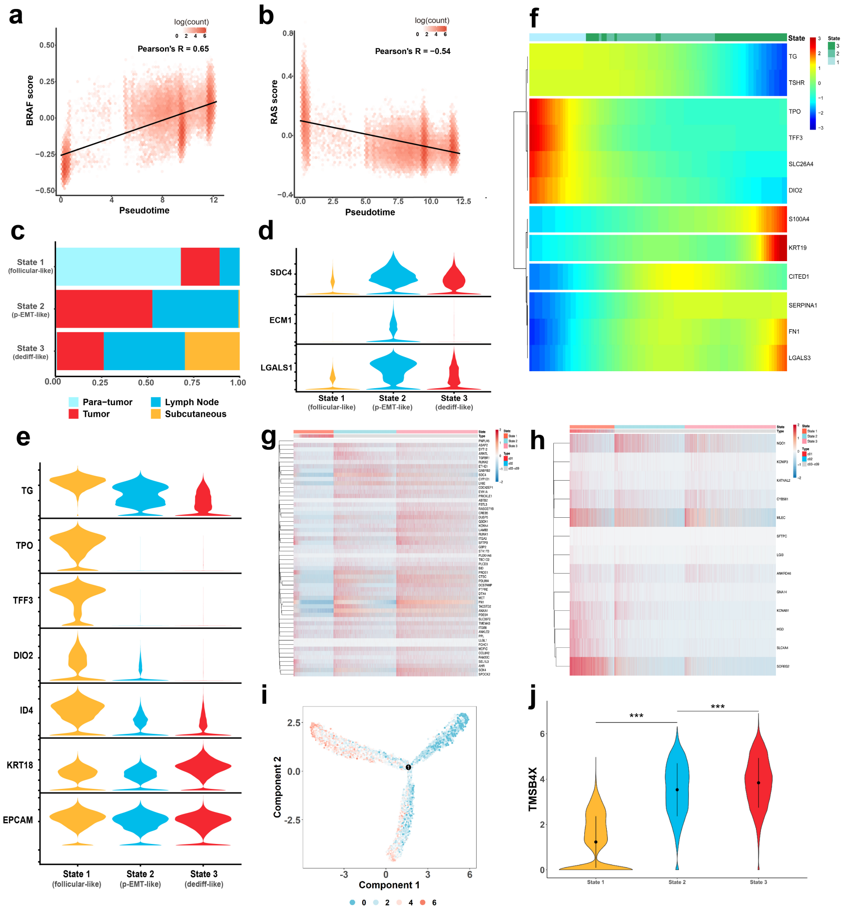

Supplementary Figure 5. Transcriptional Characterization of Three Thyrocyte States Revealed by Trajectory Analysis. a, Scatter plot showing a significantly positive correlation between *BRAF* scores and pseudotime values inferred from trajectory analysis of all thyrocytes. **b**, Scatter plot showing a significantly negative correlation between *RAS* scores and pseudotime values inferred from trajectory analysis of all thyrocytes. **c**, Different tissue origins of the three thyrocyte states. **d**, Expression level of three epithelial-mesenchymal transition (EMT)-related genes (*SDC4*, *ECM1*, *LGALS1*) in the three thyrocyte states. **e**, Violin plot showing expression level of canonical thyroid epithelial marker genes in the three thyrocyte states. **f,** Heatmap showing the expression trends of known markers. **g-h,** Heatmap showing the expression levels of *BRAF*- (**g**) and *RAS*-associated genes (**h**) in three states of thyrocytes. **i-j**, Developmental trajectory (**i**) and violin plot (**j**) showing the *TMSB4X* expression in three states of thyrocytes. In (**j**), Two-sided Wilcoxon rank sum tests were performed without multiple adjustment; ***P < 0.001. Source data are provided in the Source Data file.

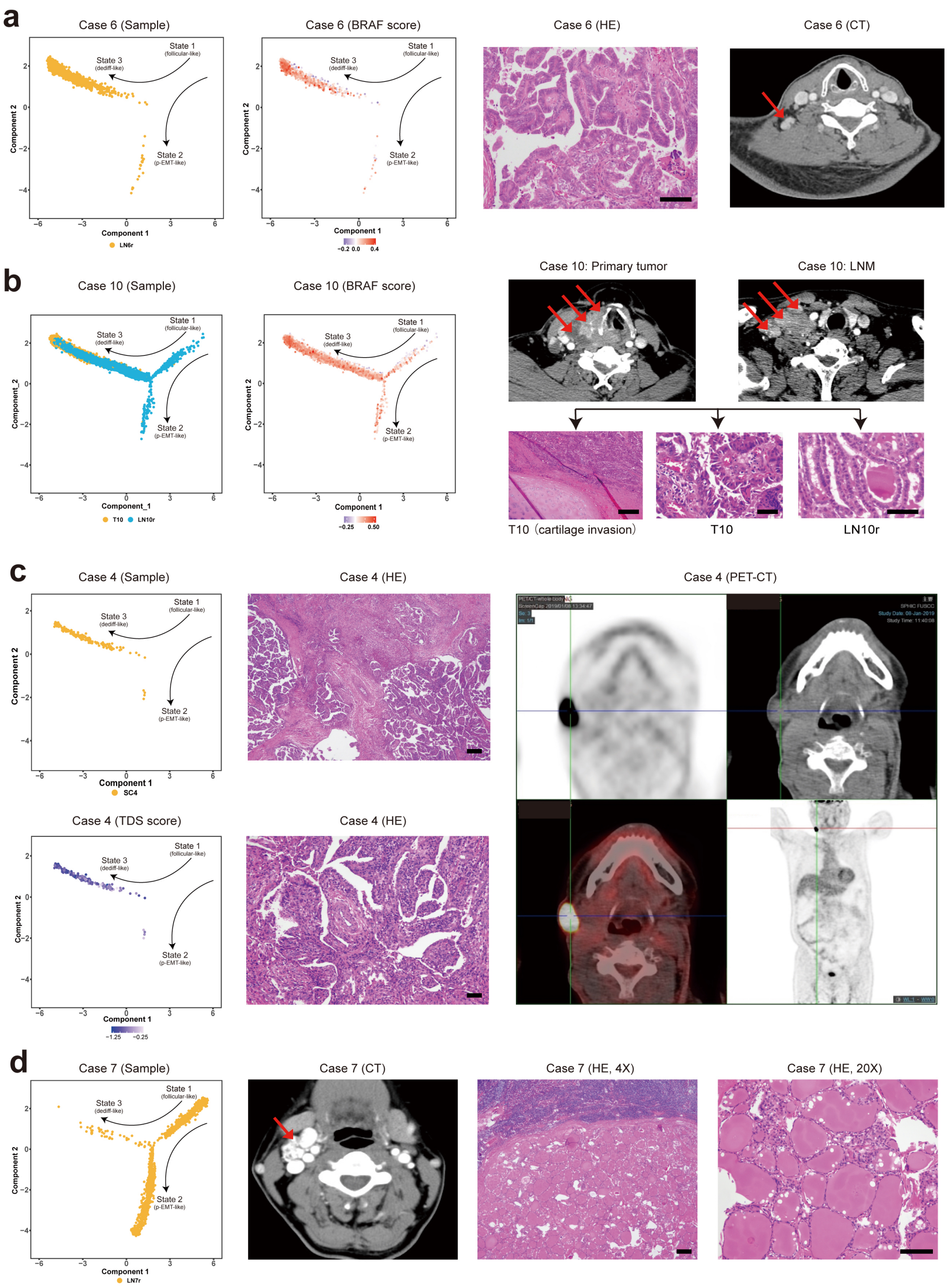

Supplementary Figure 6. Transcriptional Findings Associated with Tumor Clinicopathologic Characteristics of Four Representative Cases in Our scRNA-seq Cohort. a, H&E staining, CT scans and trajectory findings of Case 6. Scale bar = 100 µm. **b**, H&E staining, CT scans and trajectory findings of Case 10. Scale bar (left) = 200 µm; scale bar (middle) = 50 µm; scale bar (right) = 50 µm. **c**, H&E staining, PET-CT scans and trajectory findings of Case 4. Scale bar = 200 µm. **d**, H&E staining, CT scan and trajectory findings of Case 7, Scale bar (left) = 200 µm, scale bar (right) = 100 µm. In (**a**, **b**, **c**, **d**), three independent experiments were performed for each sample and generated similar results.

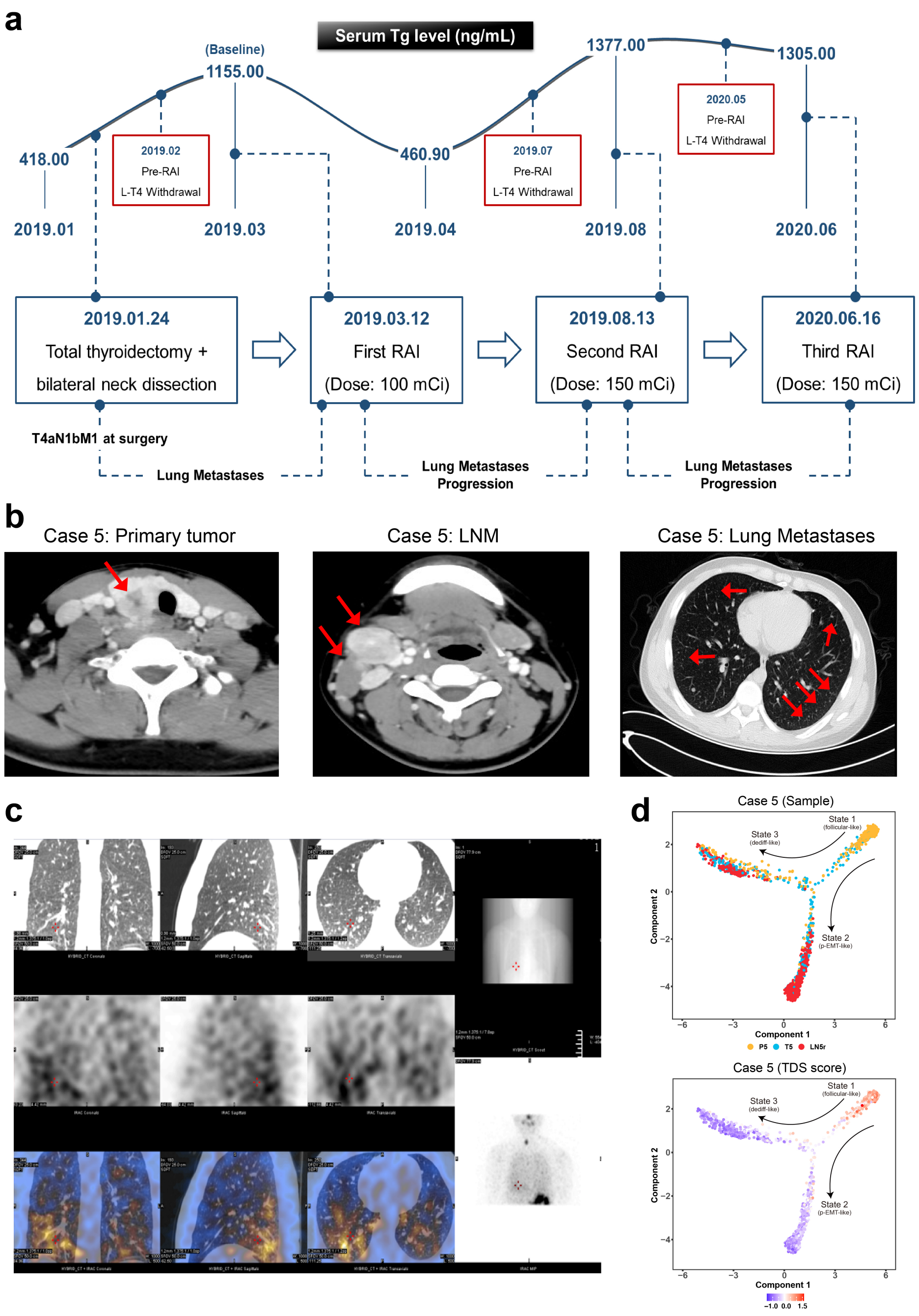

Supplementary Figure 7. Transcriptional Findings Associated with Tumor Clinicopathologic Characteristics and Responses to Radioactive Iodine Treatment of Case 5 in Our scRNA-seq Cohort. a, Clinical presentation, treatment process and follow-up of Case 5. **b**, CT scans of Case 5 at initial diagnosis (including primary tumor, lymph node metastases and lung metastases). **c**, SPECT-CT scans of Case 5 at the last follow-up showing significant progression of lung metastases. **d**, Trajectory analysis of thyrocytes from Case 5 showing a relative abundance of dediff-like (State 3) cells.

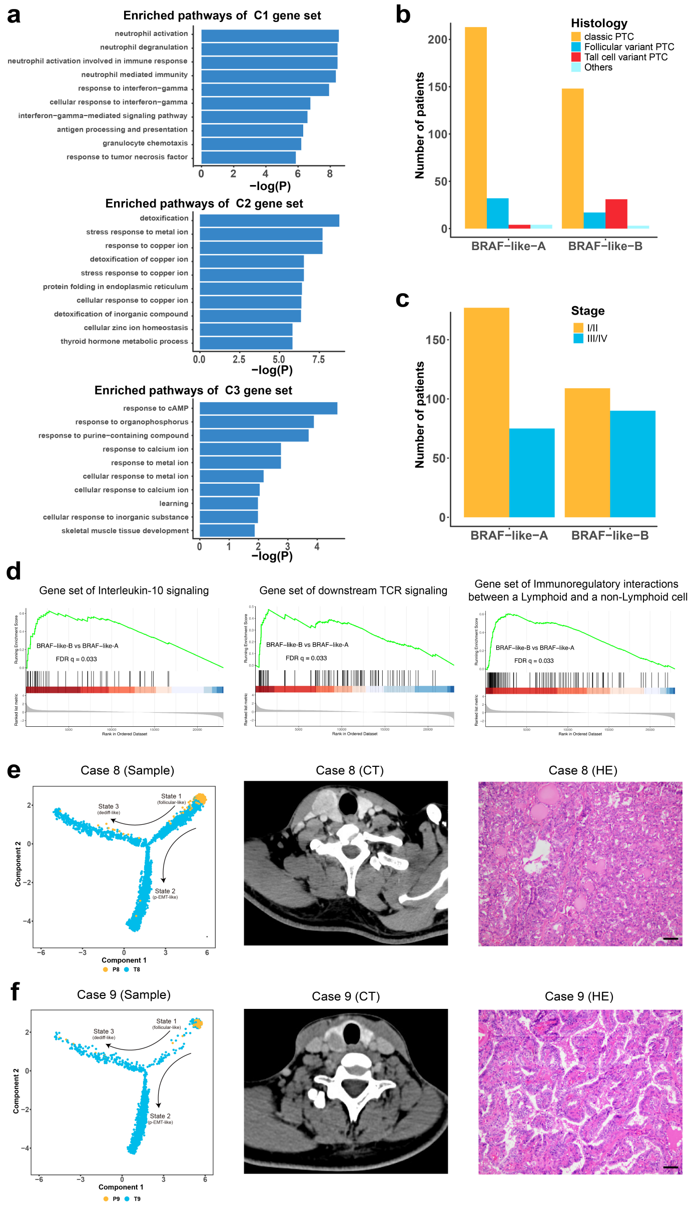

Supplementary Figure 8. Detailed Characterization of the Three Bulk Molecular Subtypes in PTCs. a, Enriched biological process (Gene Ontology) of the C1, C2 and C3 gene sets. **b**, Histogram showing the differences in histologic subtypes between *BRAF*-like-A and *BRAF*-like-B PTCs. **c**, Histogram showing the differences in AJCC stages between *BRAF*-like-A and *BRAF*-like-B PTCs. **d**, GSEA plots showing three significantly enriched immune-related pathways in the *BRAF*-like-B subgroup compared to the *BRAF*-like-A subgroup. **e**, H&E staining, CT scan and trajectory findings of Case 8. **f**, H&E staining, CT scan and trajectory findings of Case 9. In (**e**) and (**f**), scale bar = 50 μm and three independent experiments were performed for each sample and generated similar results. Source data are provided in the Source Data file.

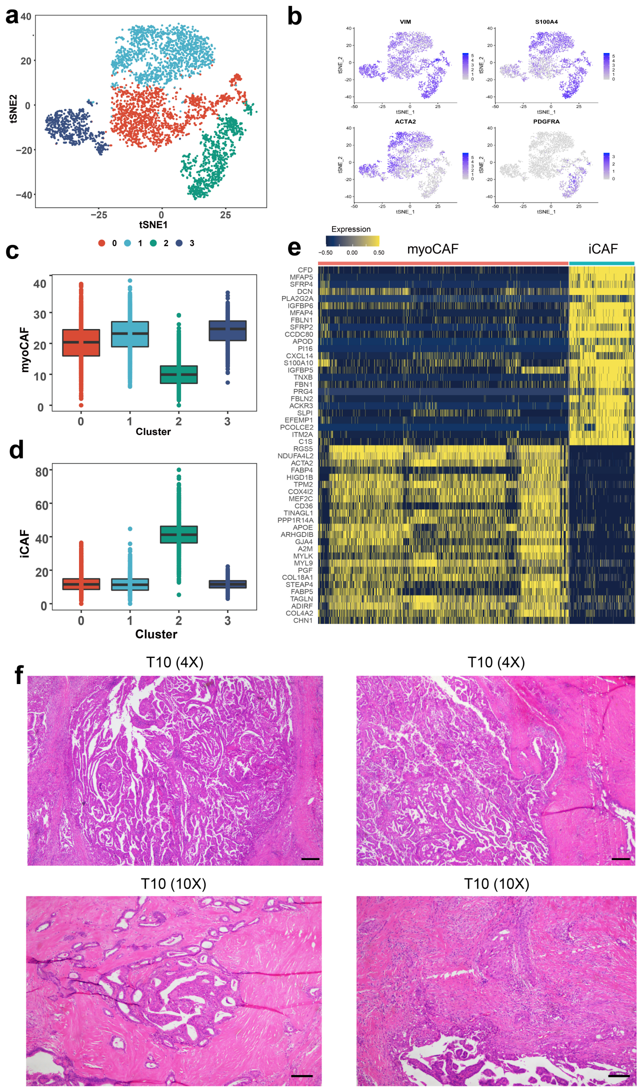

Supplementary Figure 9. Cancer-associated Fibroblasts in the PTC Ecosystem. a, t-SNE projection showing four different subclusters of CAFs with unsupervised clustering. **b**, Expression levels of known CAF markers, **c-d**, Boxplots showing the (**c**) myoCAF and (**d**) iCAF module scores for cluster 0 (n = 1780), cluster 1 (n = 1688), cluster 2 (n = 1082) and cluster 3 (n = 689). **e**, Heatmap of the top DEGs between myoCAFs and iCAFs. **f**, H&E staining showing dense desmoplasia of several fields in primary cancer of Case 10 (Magnification: 40X, upper panel; 100X, below panel). Scale bar (4X) = 200 µm; scale bar (10X) = 100 µm. Three independent experiments were performed for each sample and generated similar results. In (**c**) and (**d**), The middle lines of the boxplots show the median (central line), the lower and upper hinges show the 25-75% interquartile range (IQR), and the whiskers extend from the hinge to the farthest data point within a maximum of 1.5x IQR. Source data are provided in the Source Data file.

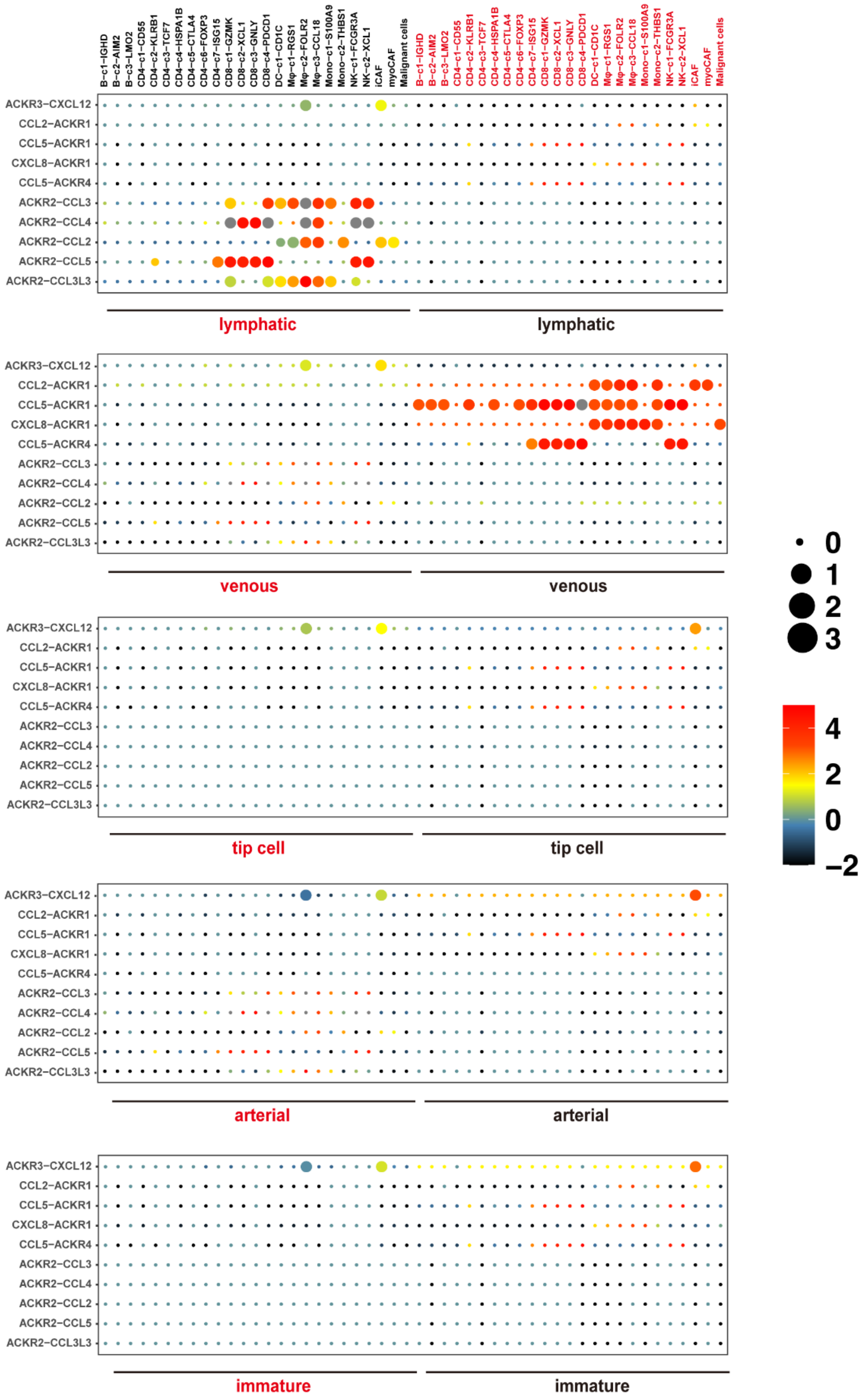

Supplementary Figure 10. Bubble plots showing the interactions between lymphatic ECs and immune cells predominantly through *ACKR2*, inferred by CellPhoneDB.

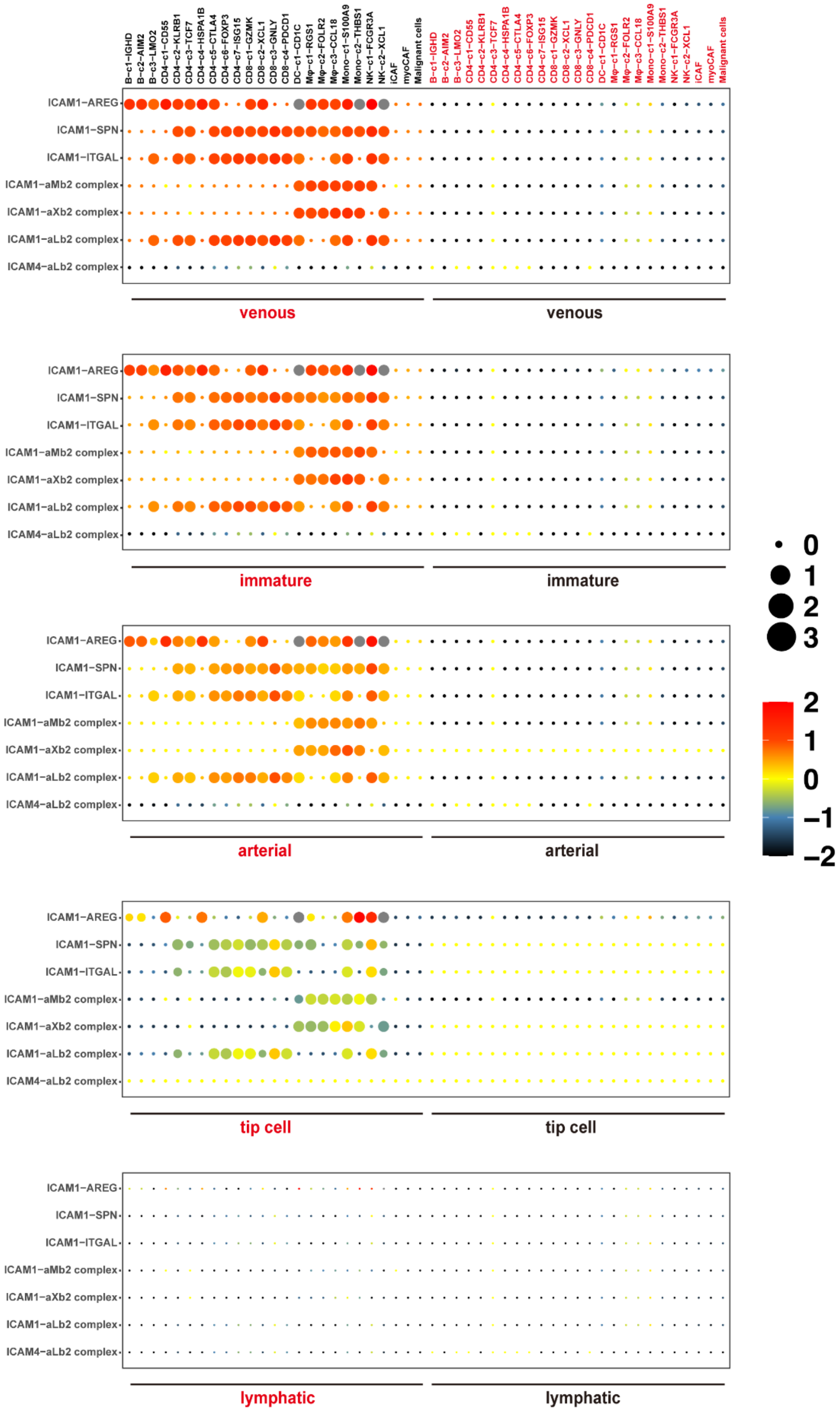

Supplementary Figure 11. Bubble plots showing the interactions of *ICAM1* and its ligands between different ECs and immune cells, inferred by CellPhoneDB.

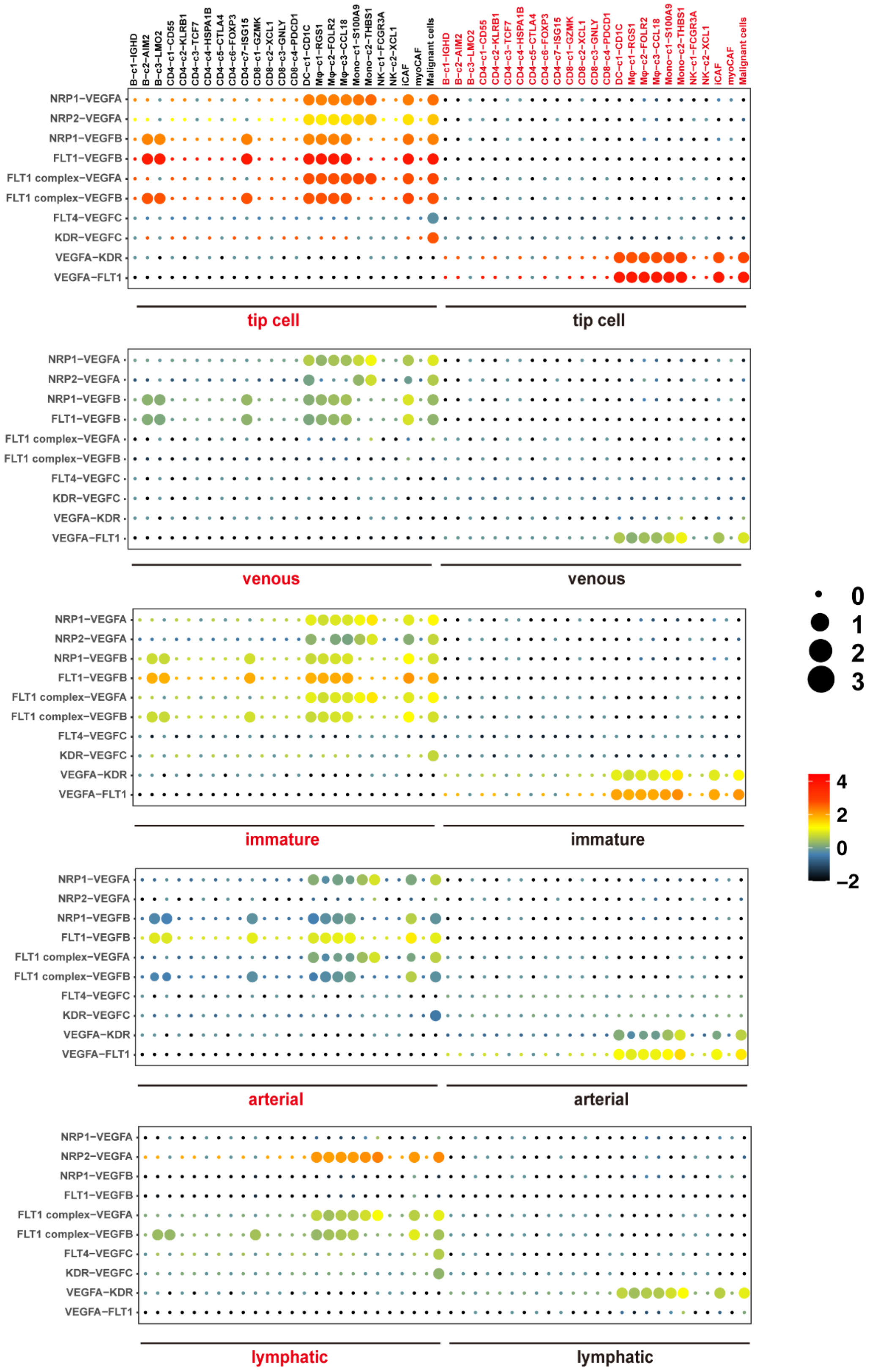

Supplementary Figure 12. Bubble plots showing VEGF-VEGFR interactions between different EC cell types and other cell types, inferred by CellPhoneDB.

Supplementary Table 1. The hotspot mutation spectrum of *BRAF*, *RAS* and *TERT* promoter detected by Sanger sequencing.

Sample ID	BRAF	TERT promoter	NRAS	KRAS	HRAS
Patient 1	V600E	WT	WT	WT	WT
Patient 2	V600E	WT	WT	WT	WT
Patient 3	WT	WT	WT	WT	WT
Patient 4	V600E	C228T	WT	WT	WT
Patient 5	WT	WT	WT	WT	WT
Patient 6	V600E	C250T	WT	WT	WT
Patient 7	WT	WT	WT	WT	WT
Patient 8	WT	WT	WT	WT	WT
Patient 9	V600E	WT	WT	WT	WT
Patient 10	V600E	WT	WT	WT	WT
Patient 11	V600E	WT	WT	WT	WT

Abbreviation: WT, wild-type.

Supplementary Table 2. Samples included in the dataset and the number of sequenced cells after quality control.

Patient ID	Sample designation	Samples collected	Sample size (cm)	Cell numbers	Median Genes
1	T1	Thyroid tumor	9.5	5331	1184
	P1	Para-tumor	N.A.	8255	736
2	T2	Thyroid tumor	2.4	4762	869
	P2	Para-tumor	N.A.	7171	1135
	LN2l	Lymph node-left	1.5	9738	983
3	T3	Thyroid tumor	1.7	6556	752.5
	P3	Para-tumor	N.A.	8404	695
	LN3l	Lymph node-left	1	8971	634
	LN3r	Lymph node-right	2.3	8703	615
4	SC4	Subcutaneous loci	2.5	2527	1176
5	T5	Thyroid tumor	2.9	3920	1060
	P5	Para-tumor	N.A.	2462	1360.5
	LN5r	Lymph node-right	3	5680	1452
6	LN6r	Lymph node-right	1.6	5290	1049
7	LN7r	Lymph node-right	1.5	7510	1101
8	T8	Thyroid tumor	2.7	8815	1566
	P8	Para-tumor	N.A.	13040	1101
9	T9	Thyroid tumor	1.7	8966	1907
	P9	Para-tumor	N.A.	6120	1462
10	T10	Thyroid tumor	4.7	8106	700
	LN10r	Lymph node-right	4.6	6418	2534
11	LN11r	Lymph node-right	6	5676	1312
	SC11	Subcutaneous loci	2.8	6156	2550

Supplementary Table 3. Overview of CD45^{+} immune cell characteristics in PTCs.

Cluster name	Representative genes	Tissue distribution (Ro/e)			
		Para-tumor	Tumor	Lymph node	Sub cutaneous
CD4-c1	CD3D, CD3E, CD3G, MT1G, MT1X, MT1E, MT2A, MT1F	0.65	0.08	1.75	0.04
CD4-c2	CD3D, CD3E, CD3G, KLRB1, IL7R, ANXA1, CD40LG, S100A11,	1.18	1.07	0.85	0.97
CD4-c3	CD3D, CD3E, CD3G, NOSIP, RGS10, LDHB, TPT1, TCF7, CCR7	1.38	0.10	1.25	0.03
CD4-c4	CD3D, CD3E, CD3G, HSPA1B, DNAJB1, HSPA1A, JUN, FOS	1.19	0.10	1.37	0.08
CD4-c5	CD3D, CD3E, CD3G, ICA1, TOX2, CD200, CTLA4, TIGIT	1.62	0.47	0.89	0.27
CD4-c6	CD3D, CD3E, CD3G, FOXP3, PDCD1, TNFRSF4, TNFRSF18, IL32, TIGIT, CTLA4	0.48	2.28	0.59	3.06
CD4-c7	CD3D, CD3E, CD3G, PLCG2, IFI44L, MX1, IFI6, STAT1	3.04	0.19	0.09	0.02
CD8-c1	CD3D, CD3E, CD3G, GZMK, CCL4, CCL4L2, GZMA, CCL5	0.89	1.45	0.78	2.17
CD8-c2	CD3D, CD3E, CD3G, XCL1, KLRC1, XCL2, GZMB, CCL5	0.67	2.51	0.45	1.45
CD8-c3	CD3D, CD3E, CD3G, GNLY, FGFBP2, NKG7, GZMH, KLRD1, GZMB	0.54	2.54	0.54	1.19
CD8-c4	CD3D, CD3E, CD3G, CCL3, CXCL13, CCL4L2, IFNG, CCL5, PDCD1	0.23	1.98	0.87	3.61
NK cell-c1	NKG7, GNLY, TYROBP, FGFBP2, FCGR3A, FCER1G, PRF1	1.06	1.13	0.75	1.11
NK cell-c2	NKG7, GNLY, TYROBP, XCL1, XCL2, KRT81, GZMK	0.84	0.64	1.70	0.69
B cell-c1	CD19, CD79A, MS4A1, TCL1A, IGHD, IL4R	0.94	0.83	1.11	0.76
B cell-c2	CD19, CD79A, MS4A1, CLECL1, AIM2, CD27	1.09	1.19	0.86	1.28
B cell-c3	CD19, CD79A, MS4A1, LMO2, LRMP	0.85	1.46	1.04	1.27
DC-c1-CD1C	CST3, LYZ, CD1C, FCER1A, CD1E, CD1A, S100B	1.05	1.02	0.96	0.80
Mφ-c1-RGS1	CST3, LYZ, RGS1, ITM2B, C3, GPR34, CD9, TM6SF1	1.13	1.21	0.40	0.14
Mφ-c2-FOLR2	CST3, LYZ, FOLR2, FCGBP, SEPP1, C1QB, C1QA, C1QC	0.78	0.97	1.19	0.42
Mφ-c3-CCL18	CST3, LYZ, CCL18, CTSL, GPNMB, CTSD, FABP5, LGALS3, MARCO	0.23	0.87	1.38	2.48
Mono-c1-THBS1	CST3, LYZ, ATP5EP2, FPR2, VCAN	0.26	1.33	0.18	0.18
Mono-c2-S100A9	CST3, LYZ, S100A9, S100A8, FCN1, S100A12, VCAN	2.16	0.65	1.78	2.08

Supplementary Table 4. Sample distribution of each thyrocyte cluster.

Sample ID	Cluster								
	c01	c02	c03	c04	c05	c06	c07	c08	c09
**LN10r**	0	0	0	5	0	3705	28	0	0
**LN11r**	0	0	0	0	0	19	1066	0	38
**LN3l**	0	0	0	6	0	0	6	6	0
**LN3r**	0	0	0	19	0	0	5	10	0
**LN5r**	0	0	0	46	0	0	107	4833	0
**LN6r**	0	0	0	5	2	0	2317	1	0
**LN7r**	1	5	1460	14	1	0	9	1	0
**P1**	2	1	0	0	0	0	5	0	0
**P3**	12	14	0	0	0	0	0	0	0
**P5**	1	1036	0	57	1	0	20	39	0
**P8**	4158	12	2	16	0	0	1	0	0
**P9**	114	0	1	1	0	0	7	0	0
**SC11**	0	0	0	1	0	4	65	0	4615
**SC4**	0	0	0	0	0	13	155	0	3
**T1**	0	0	0	0	0	0	34	0	0
**T10**	0	0	0	4	0	35	2817	0	0
**T3**	6	73	0	26	0	0	2	11	0
**T5**	5	179	2	62	0	1	23	1065	0
**T8**	53	8	27	3858	10	7	23	37	1
**T9**	300	6	5	175	3291	21	27	0	0

Supplementary Table 5. Prediction of sample type in the PRJEB11591 dataset using a machine learning classifier in this study.

Predicted Type	Actual Type	
	Para-tumor	Tumor
Para-tumor	0.97	0.04
Tumor	0.03	0.96

Supplementary Table 6. Prediction of thyrocyte cluster in our scRNA-seq data using a machine learning classifier in this study.

Type	Subcluster								
	**c01**	**c02**	**c03**	**c04**	**c05**	**c06**	**c07**	**c08**	**c09**
**Para-tumor**	0.95	0.97	0.01	0.00	0.00	0.00	0.00	0.00	0.02
**Tumor**	0.05	0.03	0.99	1.00	1.00	1.00	1.00	1.00	0.98

Supplementary Table 7. The top genes that are correlated with TDS score among thyrocytes.

Gene	Correlation
TG	0.799061298
TPO	0.784039289
TFF3	0.73892925
SLC26A7	0.738632688
SLC26A4	0.703347179
DIO2	0.670771669
IYD	0.640215968
PAX8	0.630120438
MT1F	0.626015651
SORBS2	0.623582991
MT1G	0.594213807
TSHR	0.556673962
HSP90B1	0.556343767
SORD	0.545995623
FOXE1	0.541155271
SLC26A4-AS1	0.531148797
CPQ	0.522216879
PRDX1	0.515697037
PKHD1L1	0.514920955
FCGBP	0.511997421
MATN2	0.509864677
FHL1	0.504205469

Supplementary Table 8. The most significantly differentially expressed genes between malignant and normal thyrocytes.

gene	avg_logFC	Tumor	Normal	p_val	p_val_adj
S100A4	3.39410394	0.858	0.195	0.00e+00	0.00e+00
FN1	3.35722364	0.857	0.098	0.00e+00	0.00e+00
TMSB4X	2.47482171	0.978	0.548	0.00e+00	0.00e+00
APOE	2.45766925	0.575	0.07	0.00e+00	0.00e+00
CXCL14	2.24176384	0.273	0.007	0.00e+00	0.00e+00
TIMP1	2.15109885	0.92	0.135	0.00e+00	0.00e+00
LGALS3	2.04643241	0.903	0.259	0.00e+00	0.00e+00
IGFBP6	2.0261013	0.66	0.04	0.00e+00	0.00e+00

The "p_val" column showed the pvalues calculated using the two-sided wilcoxon rank sum test, and the "p_val_adj" column showed the FDR adjusted pvalues.

Supplementary Table 9. The most significantly differentially expressed genes between premalignant and normal thyrocytes.

gene	avg_logFC	Premalignant	Normal	p_val	p_val_adj
TMSB4X	1.702889007	0.964	0.427	0.00e+00	0.00e+00
SNHG25	1.514304863	0.896	0.408	0.00e+00	0.00e+00
SNHG19	1.50952446	0.704	0.049	0.00e+00	0.00e+00
MIF	1.202374562	0.915	0.611	0.00e+00	0.00e+00
SLC25A6	1.07766644	0.902	0.584	0.00e+00	0.00e+00
C6orf48	1.011982348	0.747	0.302	3.98e-284	9.87e-280
CRIP1	1.206811368	0.624	0.217	5.27e-239	1.31e-234
PLCG2	-1.737663533	0.099	0.848	0.00e+00	0.00e+00
CD81	-1.35707445	0.27	0.878	0.00e+00	0.00e+00
IYD	-1.176065173	0.619	0.959	0.00e+00	0.00e+00
HSP90B1	-1.126684284	0.869	0.989	0.00e+00	0.00e+00
GNAS	-1.060021755	0.806	0.985	0.00e+00	0.00e+00
NEAT1	-1.110285691	0.609	0.94	2.58e-287	6.40e-283
SYNE2	-1.135044571	0.299	0.812	6.45e-286	1.60e-281
MXRA7	-1.093050496	0.21	0.755	9.45e-274	2.34e-269
DDX17	-1.042549593	0.429	0.865	1.18e-271	2.93e-267
JUND	-1.075223827	0.355	0.808	3.49e-244	8.65e-240
PKHD1L1	-1.182682878	0.035	0.559	9.14e-236	2.27e-231
VEGFA	-1.028889644	0.266	0.74	1.54e-221	3.81e-217
CRYBG3	-1.099158053	0.12	0.614	7.27e-213	1.80e-208

The "p_val" column showed the pvalues calculated using the two-sided wilcoxon rank sum test, and the "p_val_adj" column showed the FDR adjusted pvalues.

Supplementary Table 10. The distribution of the thyrocytes clusters in each thyrocyte state.

Cluster	State 1	State 2	State 3
c01	4649	0	3
c02	1317	1	16
c03	593	868	36
c04	982	2331	982
c05	31	3148	126
c06	284	114	3407
c07	7	76	6634
c08	9	5741	253
c09	1	75	4581

Supplementary Table 11. Reported markers for iCAFs and myoCAFs.

iCAFs markers	myoCAFs markers
PLA2G2A	ACTA2
MCL1	TAGLN
S100A10	MMP11
LMNA	MYL9
UAP1	HOPX
DPT	POSTN
ABL2	TPM1
EFEMP1	TPM2
TNFAIP6	IGFBP7
FBLN2	CST1
CCDC80	GRP
FSTL1	COL10A1
PTX3	INHBA
UGDH	CALD1
CXCL8	BGN
CXCL1	CTHRC1
CXCL2	
ADH1B	
CPE	
CXCL14	
GPX3	
DUSP1	
GFPT2	
TNXB	
PI16	
PIM1	
PNRC1	
SGK1	
SOD2	
APOE	
FOSB	
HAS1	
FBLN1	
ADAMTS1	

Supplementary Table 12. The primers in identifying the *RAS* mutations using Sanger sequencing.

Primer Type	Mutation	Type	Sequence
Amplification Primers	KRAS-G12	Forward	GGTACTGGTGGAGTATTTGATAG
	KRAS-G12	Reverse	ATAACTTGAAACCCAAGGTACA
	KRAS-Q61	Forward	ATGGGTATGTGGTAGCATCTCAT
	KRAS-Q61	Reverse	GCATGGCATTAGCAAAGACTCA
	NRAS-Q61	Forward	ATGGGCTTGAATAGTTAGATGCTTA
	NRAS-Q61	Reverse	GATTCTCAATGTCAAACAACCTAAA
	HRAS-Q61	Forward	TGAACTCCCCCCACGGAAGGT
	HRAS-Q61	Reverse	ATGTCCTCAAAAGACTTGGTGT
	BRAF-V600	Forward	GCTTGCTCTGATAGGAAAATGAG
	BRAF-V600	Reverse	GTAACTCAGCAGCATCTCAGG
	TERTp-lateral	Forward	TAGGCCGATTCGACCTCTCT
	TERTp-lateral	Reverse	ACCTCGCGGTAGTGGCTGC
	TERTp-internal	Forward	AGCGCTGCCTGAAACTCGC
	TERTp-internal	Reverse	CACAGACGCCCAGGACCG
Sequencing Primers	KRAS-G12-RS	n.a.	CATGAAAATGGTCAGAGAAACCT
	KRAS-Q61-F	n.a.	ATGGGTATGTGGTAGCATCTCAT
	NRAS-Q61-FS	n.a.	TTAACCTTGGCAATAGCATTG
	HRAS-Q61-F	n.a.	TGAACTCCCCCCACGGAAGGT
	BRAF-V600-F	n.a.	GCTTGCTCTGATAGGAAAATGAG
	TERTp-RS	n.a.	ACGTGGCGGAGGGACTGG
